# Association of Inflammatory and Nutritional Biomarkers in Children with Febrile Seizures: A Case–Control Study of NLR, PNI, and CAR

**DOI:** 10.3390/diagnostics16162541

**Published:** 2026-08-12

**Authors:** Peren Perk, Suheyla Gumus Sarac, Ayse Sandıkkaya, Fulya Sıbel Ekıcı Ibrahımoglu, Muhammed Talha Karadogan, Rahime Koç, Cengız Havalı

**Affiliations:** 1 Department of Pediatric Neurology, Istanbul Basaksehır Cam and Sakura City Hospital, Istanbul 34480, Turkey; aysesandikkaya@hotmail.com (A.S.); fulya.sibel.ekici@gmail.com (F.S.E.I.); cengizhavali@gmail.com (C.H.); 2 Department of Pediatric Emergency, Bursa Dortcelik Children Hospital, Bursa 16120, Turkey; shylgumus@gmail.com; 3 Department of Pediatric Rheumatology, Istanbul Basaksehir Cam and Sakura City Hospital, Istanbul 34480, Turkey; mtalhak92@gmail.com (M.T.K.); rahime-koc@hotmail.com (R.K.)

**Keywords:** febrile seizure, neutrophil-to-lymphocyte ratio, prognostic nutritional index, C-reactive protein-to-albumin ratio

## Abstract

**Background/Objectives**: Febrile seizures (FS) are the most frequent seizure disorder in childhood. Growing evidence suggests that systemic inflammatory responses may contribute to FS pathogenesis. Easily accessible inflammatory markers derived from routine blood tests, including the neutrophil-to-lymphocyte ratio (NLR), prognostic nutritional index (PNI), and C-reactive protein-to-albumin ratio (CAR), have been investigated as potential indicators of inflammatory status. This study aimed to explore the association between these biomarkers and FS in children. **Methods**: The medical records of 401 children were retrospectively evaluated, including 200 patients diagnosed with FS and 201 age-matched controls without a history of seizures. Demographic data and laboratory parameters were obtained, and NLR, PNI, and CAR were calculated. Group comparisons were performed using appropriate statistical tests. Multivariable logistic regression was applied to determine independent factors associated with FS, and receiver operating characteristic (ROC) analysis was conducted to evaluate biomarker performance. **Results**: Compared with controls, children with FS had higher neutrophil counts, NLR, and CAR values, whereas lymphocyte counts and PNI values were lower (all *p* < 0.05). Male sex was more frequent in the FS group (62.5% vs. 47.8%, *p* = 0.004). In multivariable analysis, younger age, male sex, and increased NLR were independently associated with FS. However, CAR and PNI did not remain significant after adjustment. NLR showed the highest discriminative ability among the evaluated markers, although its diagnostic performance was limited (AUC = 0.647). **Conclusions**: NLR may reflect the inflammatory response associated with febrile seizures and could provide supportive clinical information. However, its moderate diagnostic accuracy prevents its use as a standalone diagnostic marker. Further studies are required to clarify its clinical utility.

## 1. Introduction

Febrile seizures constitute the most frequently observed seizure disorder in early childhood, affecting approximately 2–5% of children and remain one of the leading causes of emergency neurological evaluation [1]. They are characterized by seizures occurring in association with fever in children aged 6 months to 6 years who have no underlying neurological disorder and no prior history of unprovoked or neonatal seizures [1,2]. Febrile seizures are classified as simple or complex based on their duration, recurrence, and focal features [1]. Although fever is common in childhood, only a subset of children develop seizures, and accumulating evidence suggests that inflammatory responses triggered by fever may contribute to seizure susceptibility [2]. The inflammatory response accompanying febrile illness has traditionally been assessed using circulating cytokines such as IL-1β, IL-6, and TNF-α [3]. Despite their biological relevance, these biomarkers are not routinely available in clinical practice due to cost and limited accessibility. In this regard, the neutrophil-to- lymphocyte ratio (NLR) has been proposed as a simple and cost-effective indicator of systemic inflammation, reflecting changes in hematopoietic lineages during inflammatory responses [4]. The assessment of serum CRP and albumin is simple, economical, and routinely performed in clinical settings.

Recent research has examined whether commonly used laboratory indices, including NLR, RDW, and platelet count, are associated with febrile seizures [5]. Both the prognostic nutritional index (PNI) and C-reactive protein-to-albumin ratio (CAR) have been recognized as markers of inflammation and clinical outcomes in various diseases [6,7]. However, their association with febrile seizures remains poorly understood, and has not been adequately investigated.

The aim of this study was to assess the relationship of CAR, PNI, and NLR with febrile seizures and to compare these biomarkers among children with recurrent febrile seizures, children with a single febrile seizure episode, and febrile children without a history of febrile seizures.

## 2. Materials and Methods

### 2.1. Study Design and Population

This retrospective case–control study was conducted at a tertiary pediatric center by reviewing the electronic medical records of children admitted to the pediatric emergency department and pediatric neurology outpatient clinic between January 2024 and May 2026. Consecutive sampling was utilized to identify eligible participants from the source population during the study period. A total of 401 participants were included in the final analysis. The study group comprised 200 children diagnosed with febrile seizures (FS), whereas the control group consisted of 201 children, established according to standard clinical criteria based on the presence of a seizure associated with fever in the absence of central nervous system infection, metabolic disturbances, or a prior history of afebrile seizures. The control group was selected from children who presented with febrile illnesses but had no documented history of febrile or afebrile seizures. To minimize age-related confounding, controls were selected using frequency matching (group matching) based on age, ensuring a similar overall age distribution between the study and control groups.

Patients with incomplete clinical records or missing laboratory data were excluded from the study. In addition, children with chronic inflammatory diseases, autoimmune disorders, hematological diseases, immunodeficiency, malignancy, severe hepatic or renal dysfunction, chronic nutritional disorders, or any other condition that could affect inflammatory or nutritional biomarkers were not included.

Febrile seizures were clinically characterized according to established criteria. Simple febrile seizures were defined as generalized seizures lasting less than 15 min and occurring once within a 24-h period, whereas complex febrile seizures were defined by prolonged duration (≥15 min), focal features, or recurrence within the same febrile episode. Because the primary objective of this study was to evaluate inflammatory and nutritional biomarkers, simple and complex febrile seizure classifications were not included in the statistical analyses. Recurrent febrile seizures were defined as two or more documented febrile seizure episodes recorded in the medical records. Prophylactic therapy was defined as intermittent benzodiazepine administration during febrile illnesses or continuous antiseizure medication use, including phenobarbital or sodium valproate, when clinically indicated based on seizure characteristics, recurrence risk, and physician assessment according to institutional practice.

### 2.2. Data Collection and Laboratory Measurements

Clinical information and laboratory findings were extracted retrospectively from the institutional electronic health record system. Routine hematological and biochemical parameters were collected from the first blood samples obtained during the initial clinical evaluation before the initiation of specific treatment (phenobarbital p.o., sodium valproate p.o., and intermittent benzodiazepine). Clinical characteristics related to febrile illness were reviewed when available from the medical records. The potential source of infection was assessed based on documented clinical diagnoses at admission, including upper respiratory tract infections, urinary tract infections, otitis media, and other identifiable infectious conditions. However, because of the retrospective nature of the study and variability in clinical documentation, infection-related data were not consistently available for all participants and were therefore not included in the comparative statistical analyses. The maximum documented body temperature at admission was also reviewed when available; however, fever temperature measurements were not recorded in a standardized manner across the study population and were therefore not included in the final analysis. Complete blood count parameters, serum C-reactive protein (CRP), and serum albumin levels were recorded for all participants. In addition to the routinely measured laboratory variables, several inflammation- and nutrition-related indices were calculated.

Inflammatory biomarkers were generated from routinely collected laboratory data. NLR was expressed as the quotient of the absolute neutrophil count and the absolute lymphocyte count, while PNI was computed using the equation below:

PNI = serum albumin (g/L) + 5 × total lymphocyte count (10^9^/L)

The C-reactive protein-to-albumin ratio (CAR) was calculated by dividing the serum CRP concentration by the serum albumin concentration.

### 2.3. Outcome Measures

The main purpose of this investigation was to determine whether variations in NLR, PNI, and CAR were associated with the development of febrile seizures. A secondary objective was to determine the diagnostic performance of these biomarkers for discriminating children with FS from those without a history of febrile seizures.

### 2.4. Ethical Considerations

The study protocol was reviewed and approved by the Local Ethics Committee at Basaksehır Cam and Sakura City Hospital before data collection (Date: 27 June 2026, Approval No: KAEK-2026-306). Because of the retrospective nature of the study and the use of anonymized patient data, the ethics committee waived the requirement for written informed consent in accordance with national regulations and institutional policies. The study was conducted in accordance with the ethical principles outlined in the Declaration of Helsinki and complied with relevant national and institutional guidelines for research involving human participants.

### 2.5. Statistical Analysis

All statistical analyses were performed using IBM SPSS Statistics, version 26.0 (IBM Corp., Armonk, NY, USA). Continuous variables were assessed for normality using the Shapiro–Wilk test. Variables with a non-normal distribution, including CRP, were compared using the Mann–Whitney U test, whereas normally distributed variables were analyzed using Student’s *t*-test or Welch’s *t*-test, as appropriate. Continuous variables are presented as mean ± SD or median (IQR), as appropriate. Categorical variables were summarized as frequencies and percentages and compared using the chi-square test or Fisher’s exact test, as appropriate.

Univariable and multivariable logistic regression analyses were performed to identify factors associated with febrile seizures. Variables that were statistically significant in the univariable analyses or considered clinically relevant based on prior evidence were considered for inclusion in the multivariable model. Results were presented as odds ratios (ORs) with corresponding 95% confidence intervals (CIs).

The discriminative ability of NLR, PNI, and CAR for febrile seizures was assessed using receiver operating characteristic (ROC) curve analysis. The area under the ROC curve (AUC), optimal cutoff values, sensitivity, specificity, positive predictive value, and negative predictive value were calculated where appropriate. A two-tailed *p*-value < 0.05 was considered statistically significant.

## 3. Results

### 3.1. Study Population

A total of 401 children were included in the study, comprising 200 patients with febrile seizures (FS) and 201 controls without a history of FS. Within the FS cohort, 105 patients were not receiving anti-prophylaxis, whereas 95 were receiving treatment. The distribution of baseline demographic variables and clinical findings across the study population is shown in Table 1. Baseline demographic characteristics are summarized in Table 1. Age and body weight were comparable between the groups, whereas male sex was significantly more frequent in children with febrile seizures.

### 3.2. Comparison Between the Fs and Control Groups

Children with febrile seizures exhibited significantly higher inflammatory markers, including leukocyte count, neutrophil count, CRP, CAR, and NLR, whereas lymphocyte count and PNI were significantly lower than those of the control group. Serum albumin levels did not differ significantly between the groups (Table 2).

### 3.3. Comparison of Treated and Untreated Patients Within the Fs Group

Among children with febrile seizures, inflammatory biomarkers (CAR, PNI, and NLR) were comparable between treated and untreated patients. However, treated patients had higher lymphocyte counts, lower albumin levels, and experienced more recurrent febrile seizure episodes than untreated patients (Table 3).

### 3.4. Three-Group Analysis

Significant differences among the three groups were observed for leukocyte count, neutrophil count, lymphocyte count, albumin, PNI, and NLR. Post-hoc analysis showed that both treated and untreated FS groups had higher neutrophil counts and NLR values than controls, whereas no significant differences were observed between the two FS groups for CAR, PNI, or NLR.

### 3.5. Logistic Regression Analysis

In multivariable logistic regression analysis, younger age, male sex, and higher NLR remained independently associated with febrile seizures, whereas CAR and PNI were not independently associated. (Table 4).

In univariable logistic regression analysis, male sex, leukocyte count, neutrophil count, lymphocyte count, PNI, and NLR were significantly associated with febrile seizures, whereas age, body weight, CRP, albumin, and CAR were not statistically significant (Table 5). Variables that were statistically significant in the univariable analyses or considered clinically relevant based on prior evidence were considered for inclusion in the multivariable logistic regression model.

### 3.6. Receiver Operating Characteristic (Roc) Analysis

**Figure 1:**
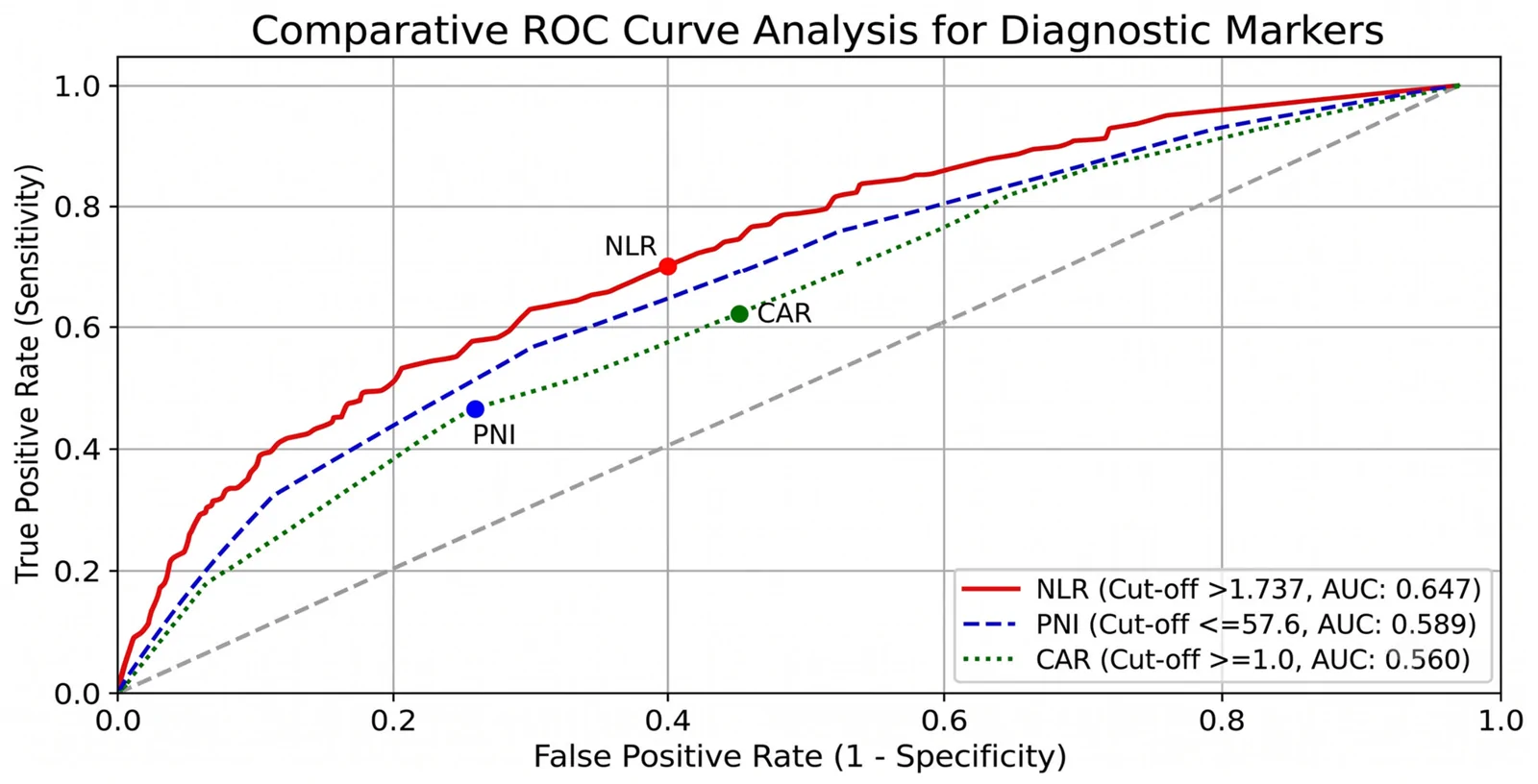
Receiver operating characteristic (ROC) curve analysis was performed to evaluate the discriminatory performance of CAR, PNI, and NLR. Optimal cut-off values were determined using the Youden index. AUC, area under the curve. The 95% confidence intervals (CIs) for the AUC were calculated using the DeLong method, whereas the 95% CIs for sensitivity and specificity were calculated using the Wilson score method. Cut-off values were estimated using the percentile method based on stratified bootstrap resampling (B = 2000). The grey dashed diagonal line indicates the reference line (AUC = 0.50) corresponding to random guessing.

ROC analysis demonstrated that NLR had the highest discriminative ability among the evaluated biomarkers, although its diagnostic performance was modest. PNI and CAR showed lower diagnostic accuracy (Figure 1).

## 4. Discussion

In this retrospective case–control study, children with febrile seizures had significantly higher NLR and CAR values and lower PNI values than controls. However, after adjustment for potential confounders, only younger age, male sex, and NLR remained independently associated with febrile seizures. Among the investigated biomarkers, NLR demonstrated the highest diagnostic performance, although its discriminative ability was modest.

The most notable observation of this analysis was that NLR remained independently associated with febrile seizures after adjustment for relevant confounding factors. Although the precise mechanisms underlying febrile seizures remain incompletely understood, increasing evidence supports a central role of inflammation in their pathogenesis [8]. Acute febrile conditions stimulate innate immune mechanisms, promoting the production of key inflammatory mediators, including IL-1β, IL-6, and TNF-α, which are thought to influence neuronal excitability. These mediators may contribute to a reduced seizure threshold by enhancing neuronal excitability and disrupting blood–brain barrier integrity [9,10].

Systemic inflammatory responses are typically accompanied by characteristic changes in peripheral leukocyte profiles. Neutrophil activation and mobilization increase during acute inflammation, while stress-related hormonal effects and immune redistribution may lead to relative lymphopenia. As a result, the neutrophil-to-lymphocyte ratio has been proposed as a simple and reliable marker reflecting the balance between innate and adaptive immune activity [11,12].

In this study, children with febrile seizures had significantly higher neutrophil counts and lower lymphocyte counts than controls, resulting in NLR values. The association remained statistically significant after multivariable adjustment for age, sex, C-reactive protein-to-albumin ratio (CAR), and prognostic nutritional index (PNI). These findings suggest that NLR may reflect the underlying inflammatory state associated with febrile seizures rather than being a secondary consequence of fever alone [13].

These observations agree with earlier clinical investigations reporting increased NLR values among pediatric patients with febrile seizures. Higher NLR values have also been associated with complex febrile seizures, further supporting the role of systemic inflammation in seizure susceptibility [14]. In the present analysis, PNI values were lower in the febrile seizure group than febrile controls in unadjusted comparisons, indicating a possible association between febrile seizures and a disturbed immune–nutritional state. Given that PNI integrates serum albumin concentration and lymphocyte count, it is generally regarded as a composite indicator reflecting both nutritional reserves and systemic immune activity [15,16].

Although PNI was significantly lower in children with febrile seizures in the univariate analysis, this association disappeared after multivariable adjustment, suggesting that PNI primarily reflects systemic inflammatory changes rather than serving as a discriminating marker of febrile seizures. Because PNI incorporates both serum albumin and lymphocyte count, its predictive contribution may overlap with NLR [15,16,17].

In this study, CAR was elevated in children with febrile seizures in univariate analysis; however, this association did not persist after multivariable adjustment, and CAR demonstrated only modest discriminative performance in ROC analysis. These findings suggest that CAR mainly reflects generalized systemic inflammation during febrile illness rather than seizure-specific mechanisms, limiting its independent clinical utility [16,17].

The analysis suggested that febrile seizures occurred more frequently in males, whereas the risk progressively declined as age increased. These findings are consistent with the well-established epidemiological pattern of febrile seizures, which predominantly occur between 6 months and 5 years of age and show a slight male predominance. The higher susceptibility in early childhood is thought to be related to the immaturity of the central nervous system and age-dependent immune responses, whereas the gender difference may reflect biological and immunological variations between sexes [18,19].

Overall, the inflammatory markers evaluated in this study showed varying degrees of clinical relevance. NLR demonstrated the strongest diagnostic performance among the tested biomarkers; however, its ability to distinguish febrile seizure cases from febrile controls remained only moderate, with an AUC of 0.647. The NLR cut-off value of 1.737 identified in our ROC analysis should not be interpreted as a universal reference value, as no established pediatric normal threshold exists. This threshold was internally derived from our study cohort and requires external validation in independent prospective populations before clinical application. This suggests that while NLR reflects underlying systemic inflammation, it is better suited as a supportive indicator rather than a standalone diagnostic tool. In contrast, PNI was significantly lower in the febrile seizure group in the univariate analysis, suggesting a possible association with combined inflammatory and nutritional status; however, this relationship did not persist in the multivariable analysis, indicating that its effect may largely overlap with other inflammatory parameters, such as NLR. Similarly, CAR was elevated in unadjusted comparisons but lost statistical significance after adjustment and showed limited discriminative ability, likely reflecting generalized acute-phase responses rather than seizure-specific pathophysiology. Taken together, these findings suggest that although all three markers are influenced by systemic inflammation during febrile illness, none of them independently provides strong discriminatory power for febrile seizures. Additionally, no meaningful differences were detected in biomarker levels between patients with and without recurrence; however, recurrence was more frequent among treated patients. This is most plausibly explained by clinical management patterns, as children with more severe or recurrent episodes are more likely to receive treatment and closer follow-up, rather than a direct influence of therapy on inflammatory profiles.

Importantly, the methodological strengths of this study include a relatively large sample size (401 patients), the simultaneous evaluation of multiple inflammatory indices (NLR, PNI, and CAR), and the use of multivariable logistic regression and ROC curve analysis to assess both independent and diagnostic performance. In addition, recalculating biomarkers directly from raw laboratory values enhances data accuracy and methodological reliability, reducing the risk of reporting or transcription bias.

The present study has several limitations that should be considered when interpreting the findings. First, its retrospective, single-center design may limit the generalizability of the results to broader pediatric populations. In addition, the analysis relied on a single laboratory measurement obtained at admission, which may not fully capture dynamic changes in inflammatory status over time. The absence of cytokine profiling, including interleukin-6 and TNF-α, also limits a more detailed understanding of the underlying immunological mechanisms. Furthermore, residual confounding cannot be completely ruled out despite multivariable adjustment. Finally, although NLR, PNI, and CAR showed measurable associations, their overall diagnostic performance remained moderate, highlighting the need for caution when interpreting these biomarkers in clinical practice.

Several clinical variables that could potentially influence systemic inflammatory dynamics, including the exact duration of fever before admission, fever characteristics, infection sources, the time elapsed between seizure onset and venipuncture, and prior medication use (e.g., antipyretics or antibiotics), could not be systematically evaluated due to incomplete and variable documentation in the retrospective records. Although blood samples were obtained during the initial clinical evaluation before inpatient therapeutic interventions to minimize temporal variability, residual confounding related to pre-hospital clinical factors cannot be completely excluded. Subgroup analyses according to infection type and detailed seizure characteristics could not be performed because of incomplete and heterogeneous retrospective documentation.

## 5. Conclusions

Although NLR, PNI, and CAR were associated with febrile seizures in univariate analyses, only NLR remained discriminating marker febrile seizures after multivariable adjustment. However, the modest diagnostic performance of NLR (AUC = 0.647) indicates that it should be regarded as a complementary inflammatory marker rather than a standalone diagnostic tool. Future well-designed prospective multicenter investigations are needed to validate these findings and clarify the clinical utility of these biomarkers.

## Figures and Tables

**Table 1:** Demographic Characteristics of Patients vs. Controls.

Parameters	Patients	Controls	*p*
Age (months)	27.48 ± 18.88	29.97 ± 18.55	0.167
Weight (kg)	14.37 ± 6.34	14.02 ± 5.12	0.878
Gender			
Female (*n*, %)	75 (37.5%)	105 (52.2%)	* 0.004
Male (*n*, %)	125 (62.5%)	96 (47.8%)	

* Data are presented as mean ± standard deviation (SD) or number (%). *p* values were calculated using Student’s *t*-test for continuous variables (age, weight) and the Chi-square test for categorical variables (gender). Abbreviations: SD, standard deviation. Bold values indicate statistically significant results (*p* < 0.05).

**Table 2:** Comparison of laboratory findings between children with febrile seizures and controls.

Parameters	Patients	Controls	*p*
CAR	1.98 (0.47–5.48)	0.95 (0.22–5.26)	* 0.037
PNI	58.72 (53.45–67.55)	62.60 (56.20–72.20)	* 0.002
NLR	2.73 (1.33–4.92)	1.46 (0.73–3.00)	* <0.001
Leukocyte Count, (×10^3^/mm^3^)	11.61 (8.82–15.88)	10.72 (8.02–13.77)	* 0.013
Neutrophil Count, (×10^3^/mm^3^)	7.25 (4.73–10.90)	5.17 (3.27–8.33)	* <0.001
Lymphocyte Counts, (×10^3^/mm^3^)	2.71 (1.77–4.43)	3.50 (2.20–5.16)	* 0.003
CRP level, mg/L	8.90 (2.18–24.05)	4.20 (1.00–23.90)	* 0.035
Albumin, g/L	45.00 (43.00–47.00)	45.00 (44.00–47.00)	0.482

* Data are presented as median (interquartile range, IQR). Group comparisons were performed using the Mann–Whitney U test because these variables were not normally distributed. Although mean ± SD values are also shown for descriptive purposes, statistical comparisons were based on the Mann–Whitney U test. *p* < 0.05 was considered statistically significant. Bold values indicate statistically significant results (*p* < 0.05). Abbreviations: CAR, C-reactive protein-to-albumin ratio; PNI, Prognostic Nutritional Index; NLR, neutrophil-to-lymphocyte ratio; CRP, C-reactive protein; IQR, interquartile range.

**Table 3:** Inflammatory and Nutritional Indices of Study Groups.

Variable	Untreated	Treated	*p*
CAR	1.98 (0.40–4.11)	2.16 (0.51–5.96)	0.241
PNI	57.20 (53.45–66.10)	60.35 (53.90–69.35)	0.384
NLR	2.92 (1.33–5.97)	2.65 (1.41–4.16)	0.101
FS episode number	1 (1–2)	2 (2–3)	* <0.001

* Continuous variables are presented as median (IQR) and were compared using the Mann–Whitney U test. Mean ± SD values are additionally provided for descriptive purposes. *p* < 0.05 was considered statistically significant. Bold values indicate statistically significant results (*p* < 0.05).

**Table 4:** Multivariable logistic regression analysis for factors associated with febrile seizures.

Predictor	OR	95% CI	*p*	Interpretation
Age (months)	0.806	0.696–0.932	* **0.004**	Independently associated
Male sex	1.752	1.162–2.641	* **0.007**	Independently associated
CAR	1.001	0.980–1.022	0.947	Not independently associated
PNI	0.987	0.967–1.007	0.196	Not independently associated
NLR	1.118	1.029–1.215	* **0.009**	Independently associated

* Multivariable logistic regression analysis was performed to identify factors independently associated with febrile seizures. Odds ratios (ORs) with 95% confidence intervals (CIs) are presented. Variables included in the model were age, sex, CAR, PNI, and NLR. Statistical significance was defined as *p* < 0.05. Bold values indicate statistically significant results (*p* < 0.05). Abbreviations: CAR, C-reactive protein-to-albumin ratio; PNI, Prognostic Nutritional Index; NLR, neutrophil-to-lymphocyte ratio.

**Table 5:** Univariable logistic regression analysis of factors associated with febrile seizures.

Predictor	β	SE	OR	95% CI	*p*
Age (months)	−0.086	0.064	0.918	0.809–1.041	0.183
Male gender	0.600	0.203	1.823	1.224–2.715	* 0.003
Weight	0.011	0.017	1.011	0.977–1.046	0.546
CRP level	0.001	0.002	1.001	0.996–1.006	0.618
Leucocyte count	0.051	0.019	1.052	1.013–1.093	* 0.009
Neutrophil count	0.086	0.023	1.090	1.041–1.141	* <0.001
Lymphocyte count	−0.116	0.047	0.890	0.812–0.976	* 0.014
Albumin	0.000	0.025	1.000	0.952–1.050	0.999
CAR	0.005	0.010	1.005	0.986–1.025	0.580
PNI	−0.019	0.008	0.981	0.965–0.998	* 0.026
NLR	0.108	0.034	1.114	1.041–1.191	* 0.002

* Univariable logistic regression analysis was performed to assess the unadjusted association between each predictor and febrile seizures. Odds ratios (ORs) with 95% confidence intervals (CIs) are presented. An OR > 1 indicates higher odds of febrile seizures, whereas an OR < 1 indicates lower odds. *p* < 0.05 was considered statistically significant. Bold values indicate statistically significant results (*p* < 0.05). Abbreviations: CAR, C-reactive protein-to-albumin ratio; PNI, Prognostic Nutritional Index; NLR, neutrophil-to-lymphocyte ratio.

## Data Availability

The data presented in this study are available on request from the corresponding author due to privacy, legal, and ethical restrictions.
